# The APSES Gene *MrStuA* Regulates Sporulation in *Metarhizium robertsii*

**DOI:** 10.3389/fmicb.2018.01208

**Published:** 2018-06-05

**Authors:** Wenjing Yang, Hao Wu, Zhangxun Wang, Qian Sun, Lintao Qiao, Bo Huang

**Affiliations:** ^1^Anhui Provincial Key Laboratory of Microbial Pest Control, Anhui Agricultural University, Hefei, China; ^2^School of Plant Protection, Anhui Agricultural University, Hefei, China; ^3^School of Transportation Engineering, Anhui Sanlian University, Hefei, China

**Keywords:** virulence, conidium capacity, transcription factor, asexual development gene, *Metarhizium robertsii*

## Abstract

The APSES family is a unique family of transcription factors with a basic helix-loop-helix structure (APSES: *Asm1p, Phd1p, Sok2p, Efg1p*, and *StuAp*), which are key regulators of cell development and sporulation-related processes. However, the functions of the APSES family of genes in the entomopathogenic fungus *Metarhizium robertsii* have not been reported. Here, we report the identification and characterization of the *MrStuA* gene, a member of the APSES family, in *M. robertsii*. The selected gene was identified as *StuA* in *M. robertsii* (*MrStuA*) because the gene product contains two conserved sequences, an APSES-type DNA-binding domain and a KilA DNA-binding domain, and has the highest homology with the StuA in the C-II clade of the APSES family. We found that the number of conidia produced by the Δ*MrStuA* strain was 94.45% lower than that in the wild type. Additionally, in the mutant, the conidia displayed an elongated shape, the sporulation was sparse and the phialide were slender. In addition, transcription levels of two central regulators of asexual development, *AbaA* and *WetA*, were significantly reduced in the mutant; furthermore, the transcription levels of other sporulation related genes, such as *Mpk, Phi, Med, Aco, Flu*, and *FlbD*, also decreased significantly. We also show that the median lethal time (LT_50_) of the mutant increased by 19%. This increase corresponded with a slower growth rate and an earlier conidia germination time compared to that of the wild strain. However, the resistance of the mutant to chemicals or physical stressors, such as ultraviolet radiation or heat, was not significantly altered. Our results indicate that in *M. robertsii, MrStuA* may play a crucial role in regulating sporulation as well as virulence, germination, and vegetative growth. This study improves our understanding of the impact of the transcription factor StuA on sporulation processes in filamentous fungi and provides a basis for further studies aimed at improving sporulation efficiency of these fungi for use as a biocontrol agent.

## Introduction

Transcription factors are an important component of the signal transduction pathway, which regulates the expression of the target gene(s) to further regulate the growth and differentiation of cells (Shelest, 2008). There are several exclusive transcription factor families in fungi, including the TEA/ATTS and APSES families. The transcriptional regulatory proteins in the APSES family belong to the bHLH class of transcription factors. There are five types of proteins in the APSES family, *Asm1, Phd1, Sok2, Efg1*, and *StuA*; these proteins play important roles in mycelial growth, sporulation, and pathogenicity (Twumasi-Boateng et al., 2009). In recent years, a large number of APSES family proteins have been identified, especially in filamentous fungi. There are five members identified in most fungi, including *Aspergillus fumigatus* and *A. nidulans*; meanwhile, other fungi, such as *Magnaporthe oryzae* and *Cryptococcus neoformans*, have fewer members (between two and four) (Nishimura et al., 2009).

*A. nidulans StuA* was the first transcriptional factor with a basic helix-loop-helix structure found in the APSES family (Ballance et al., 1983). The gene, which has a 3-kb-long promoter that is regulated by the most prominent regulator *BrlA*, is responsible for the spatial expression of the *AbaA* transcription factor (Wu and Miller, 1997). This protein plays an important role in the structure and cell morphogenesis during the sexual and asexual phases of reproduction in *A. nidulans*. However, the transcription factors in the APSES family have different functions in different species. For example, they regulate attachment-mediated infection in *M. grisea* (Nishimura et al., 2000); yeast-mycelial two-phase transformation in *Wangiella dermatitidis*; secondary metabolites in *A. fumigates* (Twumasi-Boateng et al., 2009); bisexual dimorphism, sporulation, and pathogenic development in *Ustilago maydis* (Garcia-Pedrajas et al., 2010); and conidia development, pathogenicity, and secondary metabolites in *Fusarium graminearum* (Lysoe et al., 2011).

The STUA protein (65.01 kDa) in *A. nidulans* contains two conserved fragments: an APSES-type DNA-binding domain and a KILA (N-terminal/APSES-type HTH) DNA-binding domain (Twumasi-Boateng et al., 2009). The two conserved domains regulate the expression of related genes through interactions with the STREs sequence of the promoter region in the *AbaA* gene, thus impacting the conidiation of strains. For example, a spatial rearrangement of the conidiophores and a small amount of visible conidia are observed in the *A. nidulans* knockout mutant of *StuA*; meanwhile, no normal conidiophore structure has been found in a *Penicillium oxalicum StuA* mutant. The *StuA* gene has two transcription sites, *StuAα* and *StuAβ*. *StuAα* activates the first intron in *StuAβ*. Both of the sites have long 5^′^ leaders and mini open reading frames (ORFs) that code regulatory functions of *StuA* at a translational level.

*M. robertsii* (formerly known as *M. anisopliae*) is an important insect pathogen that is commonly used as a biological control agent (Zhou et al., 2012). Spores are the main reproductive organs of fungi as well as the main component of fungal pesticides. The bottleneck limiting the use of *M. robertsii* as a biopesticide is the low sporulation rate. Thus, a greater understanding of the function of sporulation-related genes will help make the application of strains as fungicides feasible.

There are some existing reports on sporulation-related genes in the genus *Metarhizium*. The molecular mechanism of the sporulation process is complicated in *M. anisopliae* and other entomopathogenic fungi (Clarkson and Charnley, 1996). Two sporulation pathways involving many regulatory genes, such as *Flu, G-flbs*, and *FadA*, co-regulate sporulation in *M. robertsii*; this process is similar to that of *A. nidulans*. The *Cag8* gene, which is homologous with the *FlbA* gene in *A. nidulans*, is required for sporulation. This requirement has been confirmed by showing that a knockout strain cannot produce conidia (Timberlake, 1980). Using RNA interference (RNAi), a micro-circulation sporulation gene *Pky* was found to produce more varied shapes of conidia (Xu et al., 2011).

The mechanisms of sporulation in *A. nidulans* and *Neurospora crassa* have been well studied (Park and Yu, 2012). Blasting the sequences of *StuA* and other sporulation related genes from *A. nidulans*, such as *BrlA, AbaA, WetA, FlbA, FluG, AcoB, FlbC, FlbD, MedA, PhiA, SteC, MpkA, FadA, HymA*, and *Sak A*, show that homologous genes also exist in *M. robertsii*. Thus, it is speculated that the molecular mechanism of sporulation in *M. robertsii* may be similar to that of *A. nidulans*. However, the functions of the APSES family of proteins in the entomopathogenic fungi *M. robertsii* have not been reported. We generated a *MrStuA* knockout mutant (Δ*MrStuA*) and found that *StuA* may play a crucial regulatory role in sporulation, virulence, germination, and vegetative growth in *M. robertsii*. This study deepened our understanding of the impact of the *StuA*-like transcription factor on sporulation in filamentous fungi.

## Materials and Methods

### Fungal Culture

The wild-type (WT) strain *M. robertsii* ARSEF23 (ATCC No. MYA-3075) was obtained from the Anhui Provincial Key Laboratory of Microbial Pest Control (RCEF5501). To obtain conidia, the WT strain, a *MrStuA* knockout strain (Δ*MrStuA*), and a complemented strain (C’) were grown on potato dextrose agar (PDA; 200 g of potato, 20 g of dextrose, 20 g of agar and 1,000 ml of water) at 25°C for 7–8 days. Subsequently, conidial suspensions (0.05% v/v Tween 80) were obtained by filtering the cultures through sterile non-woven fabrics to remove mycelia. The conidial suspensions were inoculated onto PDA medium overlain with sterile cellophane paper using spreaders and incubated at 25°C. Colony phenotype assays were performed on solid PDA, 1/4 Sabouraud dextrose agar with yeast extract (1/4 SDAY; 10 g of glucose, 2.5 g of yeast extract powder, 2.5 g of peptone, 20 g of agar, and 1,000 ml of water) and SDAY.

The host *E. coli* Trans 10 was purchased from the TransGen Biotech Corporation (China). The plasmid pDHt-bar/ben and *Agrobacterium tumefaciens* AGL-1 were donated by Chengshu Wang (Shanghai Institute of Plant Physiology and Ecology, Chinese Academy of Sciences).

### Peptide Domain and Homology Analysis

**FIGURE 1:**
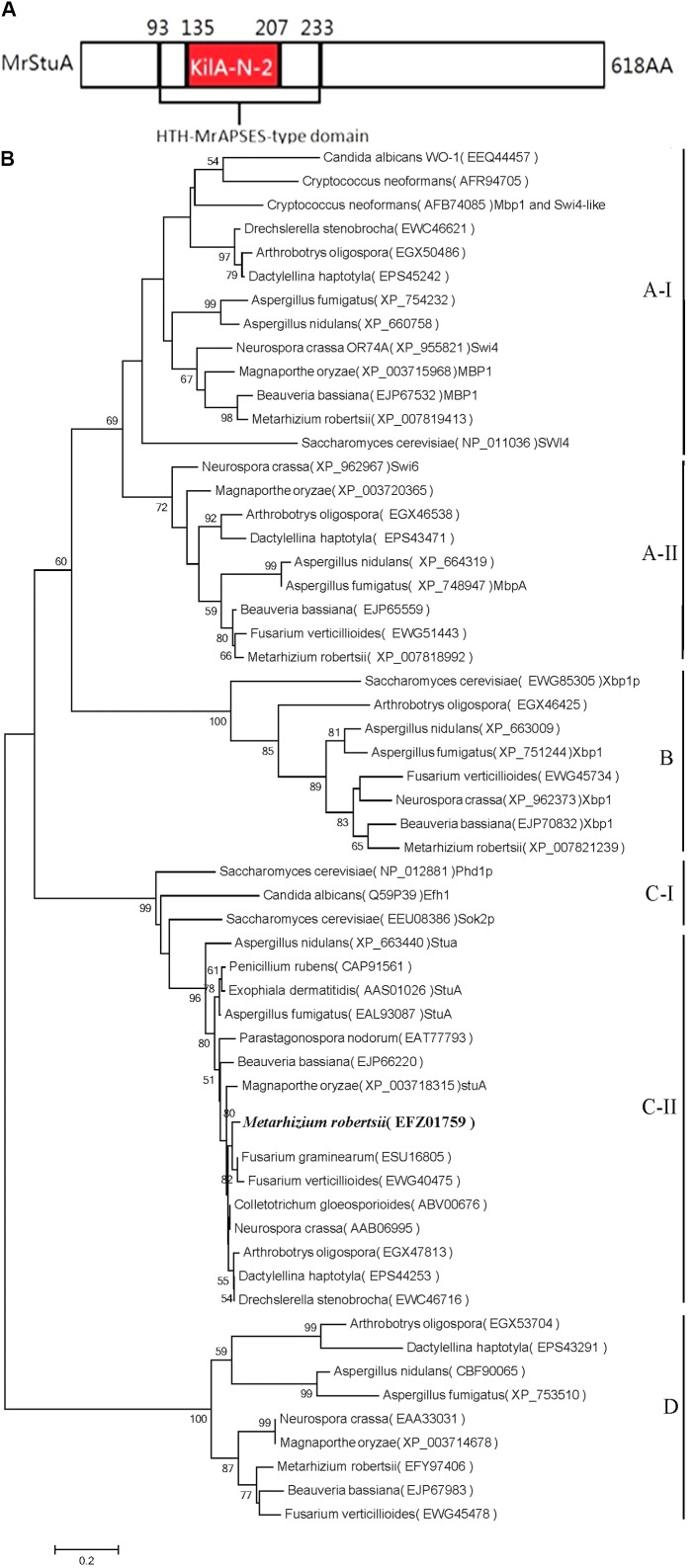
Bioinformatics analysis of the *MrStuA* protein. **(A)** Peptide domain analysis of the APSES transcription factor from *M. robertsii*. **(B)** A phylogenetic tree based on the APSES-type DNA-binding domains from different fungi. Bootstrap values (expressed as percentages of 1000 replications) greater than 50% are indicated on the tree. Numbers below the nodes indicate the bootstrap values. The *bar* markers indicate genetic distances, which are proportional to the number of amino acid substitutions.

The amino acid sequences of APSES proteins were retrieved from NCBI^1^. The conserved domains were predicted using InterProScan 5.0. We identified the protein structure of the ARSEF 23 APSES transcription factor (XP_007819177) (**Figure 1A**) and found that this protein has high homology with the APSES family of proteins from a diverse range of fungi.

### Multiple Alignment of the APSES-Type DNA-Binding Domain and Construction of a Phylogenetic Tree

The conserved amino acid sequences of the APSES domains (IPR003163) were aligned using Clustal W parameters (**Supplementary Figure S1**). MEGA version 5.1 was used to construct a neighbor-joining (NJ) tree with bootstrap analysis using 1,000 replicates. In total, 58 APSES domains from different fungi were identified for phylogenetic analysis. The neighbor-joining tree was submitted to TreeBase (**Figure 1B**) (Study Accession URL^2^).

### Construction of a *MrStuA* Knockout Mutant, Δ*MrStuA*

To characterize the functions of the *MrStuA* in *M. robertsii*, a gene replacement mutant was generated using a dominant selectable marker gene, *bar*. The Δ*MrStuA* deletion mutant was constructed by replacing the *MrStuA* gene regions with the *bar* gene cassette. A complementation strain was obtained by transforming pben-MrStuA into the Δ*MrStuA* strain. Based on the gene sequences of APSES (MAA_02988) transcription factors in *M. robertsii* ARSEF 23, the primers *MrStuA*-F and *MrStuA*-R (**Supplementary Table S1**) were designed to amplify the *MrStuA* fragment. The PCR amplification product was purified, cloned, and sequenced.

A *MrStuA* deletion vector, pDHt-bar-StuA-Del, was constructed by inserting two flanking sequences of *MrStuA* into the left and right sides of bar (a kanamycin resistance gene) in the pDHt-bar vector using the primers *MrStuA*upF+*MrStuA*upR (**Supplementary Table S1**). A 1,008 bp upstream flanking sequence of *MrStuA* was amplified from *M. roberstii* genomic DNA and was inserted into the EcoR I-Pst I sites of the pDHt-bar vector to generate a plasmid, pDHt-bar-StuA-upstream. Subsequently, a 1,285 bp downstream flanking sequence of *MrStuA* was amplified from *M. roberstii* genomic DNA using the primers *MrStuA*downF+*MrStuA*downR (**Supplementary Table S1**). This sequence was inserted into the XbaI site of the pDHt-bar-StuA-upstream vector to generate the pDHt-bar-StuA-Del plasmid. Finally, a 3,267 bp fragment containing the StuA- upstream- bar- StuA- downstream cassette was obtained through PCR amplification with the *MrStuA*upF+*MrStuA*downR primers from the pDHt-bar-StuA-Del vector. The resulting PCR product was purified and used for *A. tumefaciens*-mediated transformation. The Δ*MrStuA* strain was constructed using *Agrobacterium*-mediated fungal transformation (ATMT) and inserted by homologous recombination as previously described (Duan et al., 2009).

All recombinant strains were confirmed by PCR and reverse transcription-PCR (RT-PCR). PCR analysis indicated that a 1,050-bp fragment corresponding to the partial *MrStuA* gene was present in the WT and C’ strains, but not in the Δ*MrStuA* strain. A partial bar gene fragment (814-bp) was present in the Δ*MrStuA* and C’ strains, and a partial Ben gene fragment (328-bp) was present in the C’ strain.

### Construction of the Complement Plasmid for Δ*MrStuA*

The *MrStuA* complement plasmid Δ*MrStuA*::*MrStuA* was constructed using the backbone of the pDHt-ben vector. The full-length *MrStuA* (3,528 bp including the promoter region) was amplified from the genomic DNA of the WT strain using cp*MrStuA*upF+cp*MrStuA*upR primers (**Supplementary Table S1**). This sequence was subsequently cloned into the Spe I-Xba I site of pDHt-ben to create the pDHt-ben-Δ*MrStuA*::*MrStuA* plasmid. Transformation of pDHt-ben-Δ*MrStuA*::*MrStuA* with the full length of *MrStuA* was performed as previously described using carb (50 μg/mL) and kan (50 μg/mL) as selective agents. Transformants were co-cultured with the Δ*MrStuA* strain. A portion (1,050 bp) of the *MrStuA* sequence was amplified using *MrStuA*F+*MrStuA*R primers (**Supplementary Table S1**) to obtain the C’ strain.

### Morphological Observations at the Microscopic Level

The WT, Δ*MrStuA*, and C’ strains were grown on PDA medium at 25°C for 3 days to determine hyphal growth and conidiation. Fresh mycelia and conidia were taken from the edges of colonies of each strain and stained with lactophenol cotton blue. Micromorphology was examined using an Olympus optical microscope (BX51, Olympus Co, Tokyo, Japan) and measured using the Measurement Tool program in the DP2-BSW software.

### Sporulation Capacity Tests

Fresh conidia from the WT, Δ*MrStuA*, and C’ strains were collected after being cultured at 25°C for 8 days. The conidia were suspended, and 30 μl of each suspension was inoculated onto solid PDA (35 mm disposable plastic petri dish) for incubation at 25°C for 12 days (Liu et al., 2013; Xiong et al., 2013). The quantity of conidia in each plate was counted using a hemacytometer, after which the number of conidia per unit area was calculated. There were three replicates for each strain and all assays were repeated in triplicate.

### Color, Morphology, and Growth of Colonies

The WT, Δ*MrStuA*, and C’ strains were grown on PDA, SDAY, and 1/4SDAY plates, respectively, at 25°C for 7–8 days. Conidial suspensions (10^7^ conidia/mL) were prepared for each strain to inoculate a new plate (1 μl of suspension/plate). These plates were cultured at 25°C for 12 days. Each strain was inoculated on 3–5 plates. All assays were repeated in triplicate. The morphology and growth status of the colonies were observed and measured every 48 h starting on the fourth day of culture. The diameter of each colony was measured by the cross method, and all assays were repeated in triplicate.

### Changes in Germination Patterns

Conidia were harvested and suspended in Tween 80 solution (0.05% v/v). Then, 30 μL of the filtered suspension (10^6^ conidia/mL) was resuspended in 30 μL of GM (0.2% trehalose, 0.25% peptone, and 0.25% yeast extract powder) liquid medium and incubated at 25°C for 16–22 h. Germination was observed at 400× magnification after the conidia were placed on the medium. All assays were repeated in triplicate.

### Chemical Stress Challenges

To examine chemical stress tolerance, the mycelium of the WT, Δ*MrStuA*, and C’ strains were cut off from YEB medium by perforations (Chen et al., 2015a). The mycelium of each strain were grown on solid PDA and PDA supplemented with 2 μg/mL sodium dodecyl sulfate (SDS), 2 μg/mL Congo red (CR), 1 mol/L NaCl, 2 μg/mL carbendazim, 2 μg/mL menadione, and 2 mmol/L H_2_O_2_. Then, the mycelium were incubated at 25°C for 10 days. Morphological changes in the colonies were observed and measured. All assays were repeated in triplicate.

### UV Radiation and Heat Shock Assay

Thirty microliters of conidial suspensions (10^6^ conidia/mL) were placed on a glass slide and dried on a sterile table to eliminate moisture. The UV light wavelength of the hybrid furnace was set to UV 254 nm, Time1, Energy2. Germination liquid (GM) was added after UV irradiation, and samples were cultured at 25°C for 16–24 h. At the same time, the filtered suspensions were transferred to Eppendorf tubes and treated in a 42°C water bath for 1 h. Aliquots of the suspensions (30 μL) were inoculated onto PDA medium (35 mm disposable plastic petri dish) and cultured at 25°C for 16–24 h for examination of conidium germination. The data were recorded every 2 h. The inhibition rates were then analyzed (Rangel et al., 2006; Chen et al., 2015b).

### Virulence Assays

To investigate the effects of *MrStuA* on fungal virulence, insect bioassays were conducted using wax moths. Conidia harvested from cultures grown on PDA medium were applied topically by immersing the wax moths for 1 min in an aqueous suspension (10^7^ conidia/mL). Each treatment had three replicates with 15 insects each, and mortality was recorded every 12 h to calculate the median lethal time (LT_50_).

### Real-Time Quantitative Reverse Transcription PCR

Hyphal and conidia were harvested from cultures grown on solid PDA for 60 h (initial period of conidia formation). Total RNA was extracted from the samples using the Trizol reagent (Invitrogen, Foster City, CA, United States). cDNA was synthesized from 1 μg of total RNA using Moloney murine leukemia virus reverse transcriptase (TaKaRa, Dalian, China) according to the manufacturer’s instructions. This cDNA was used as the template for qRT-PCR with specific primer pairs (**Supplementary Table S2**). The qRT-PCR was performed using the SYBR Green (TAKARA) detection method (CFX96TM Real-Time System, Bio-Rad Laboratories, Hercules, CA, United States). All PCR reactions were performed in triplicate. The 2-ΔΔCT method (Livak and Schmittgen, 2001) was used to calculate relative expression levels, and the glyceraldehyde 3-phosphate dehydrogenase gene (primer pair gpdF/gpdR) was used as an internal control for each sample. The data are expressed as the mean ± standard deviation (SD) for three independent experiments.

## Results

### Summary of the Gene *StuA* in *Metarhizium robertsii*

An analysis of the *M. robertsii* genome database revealed that the putative *StuA* transcription factor gene consists of 2,074 nucleotides with two introns and three exons (427 bp, 94 bp, and 1,336 bp) and is predicted to encode a protein with 618 amino acids. The protein contains two conserved sequences, the APSES-type DNA-binding domain (IPR003163, 93rd–208th amino acid) and the KILA, N-terminal/APSES-type HTH, DNA-binding domain (IPR018004, 135th–207th amino acid, **Figure 1A**). The APSES core domain in fungi contains 73 amino acid residues consisting of six beta-folded and two pairs of alpha helices. The amino acid sequence from *M. robertsii* putative *StuA* had 65%, 43%, and 43% homologies with those of *F. graminearum, A. nidulans*, and *P. chrysogenum*, respectively.

Based on the phylogenetic tree (**Figure 1B**), the selected APSES family proteins were divided into four clades (A, B, C, and D), each of which has differently conserved amino acid sequences. The *M. robertsii* putative *StuA* transcription factor gene (XP_007819177) is in the C clade, which also includes the *StuA* genes from *F. graminearum, A. nidulans, P. chrysogenum*, and *Beauveria bassiana*. Within the clade, the *M. robertsii* gene is most closely related to the homologous genes from *F. graminearum* and *F. verticillioides* and is most distinct from that of *B. bassiana*. Based on the conserved domains, homology with other genes, and similarity within the phylogenetic tree, the gene was identified as the *StuA* transcription factor and was named *MrStuA*.

### *MrStuA* Is Required for Sporulation

**FIGURE 2:**
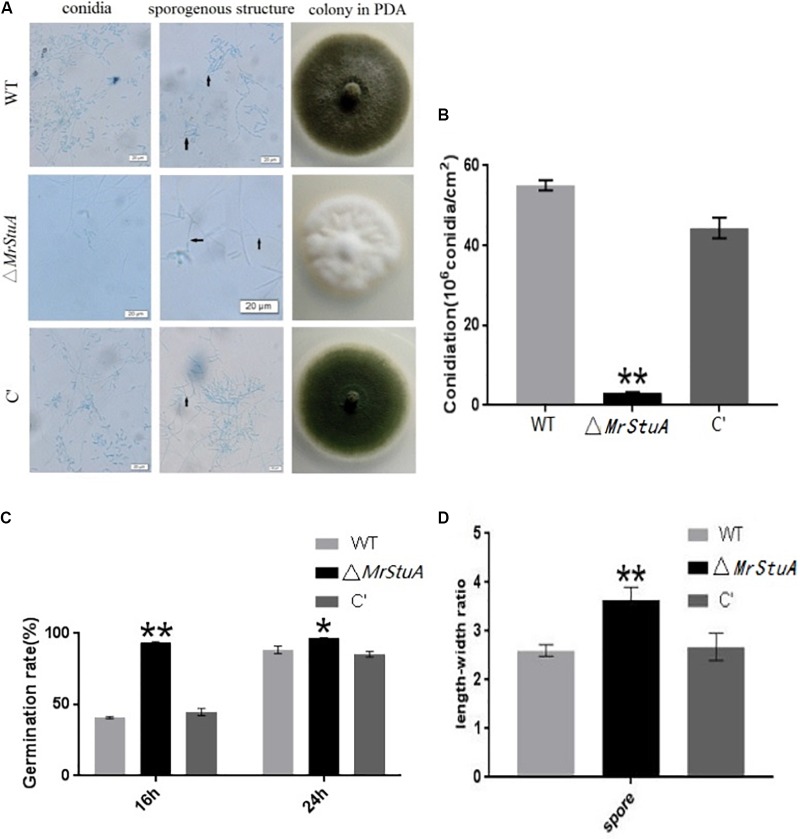
Changes in conidia capacity, conidium shape, conidiophore structure, and germination time between the wild type and mutant strains. **(A)** Morphology of the conidia, hypha and conidiophore structures, and colonies for the WT, Δ*MrStuA*, and C’ strains. **(B)** Conidia numbers per growth area (cm^2^) for the WT, Δ*MrStuA*, and C’ strains. **(C)** Germination rate of conidia for the WT, Δ*MrStuA*, and C’ strains. **(D)** The aspect ratio of the conidia for the WT, Δ*MrStuA*, and C’ strains. Student’s *t*-test: ^∗^*p* < 0.05, ^∗∗^*p* < 0.01.

There were numerous morphological differences between the colonies of the Δ*MrStuA* strain and those of the WT and C’ strains. The Δ*MrStuA* strain formed white colonies with only a few conidia; meanwhile, the WT and C’ strains produced dark green colonies with many green conidia (**Figure 2A**). On PDA medium, the Δ*MrStuA* strain yielded 94.45% fewer conidia than did the WT strain, and the mutant was nearly totally unable to produce conidia (**Figure 2B**). Microscopic observations showed that the conidium shape, sporulation structure, and phialides were elongated, sparser, and more slender, respectively, in the mutant compared to those in the wild type. In the wild-type and C’ strains, the conidia column contained many parallel conidial chains; however, this feature was not present in the mutant. A length–width ratio was calculated for conidia to better determine the variety in conidia shape. This ratio revealed that the Δ*MrStuA* strain (ratio = 3.64 ± 0.36) had conidia that were significantly more slender (40%) than those of the WT strain (ratio = 2.6 ± 0.36) (*P* < 0.01) (**Figure 2D**).

After 16 h, the germination rate of the Δ*MrStuA* strain was more than 90%; meanwhile, the germination rates of the WT and C’ strains were less than 50%. However, there were no differences in germination rate between strains at 24 h (**Figure 2C**). Thus, it appears that germination occurs sooner in the mutant strain than that in the WT or C’ strains.

Deletion of MrStuA led to changes in sporulation capability, conidia shape, conidiophore structure, and germination time; thus, it appears that MrStuA is required for sporulation.

### Expression of Sporulation Related Genes in Δ*MrStuA*

**FIGURE 3:**
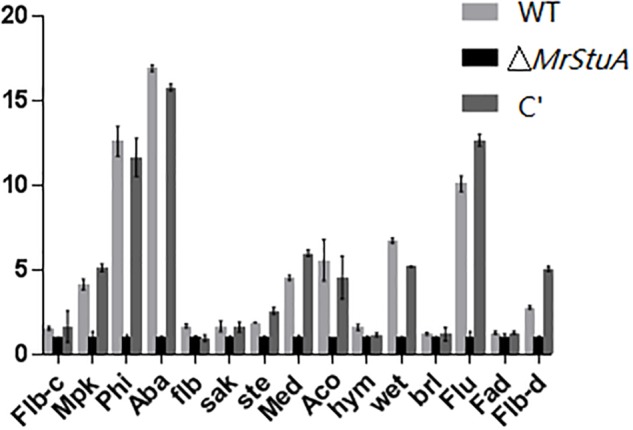
qRT-PCR of the expression of sporulation related genes in the Δ*MrStuA* strain.

We monitored the mRNA levels of genes involved in conidiogenesis in *M. robertsii* by qRT-PCR, including the *FlbA, BrlA, FluG, AcoB, AbaA, FlbC, FlbD, MedA, PhiA, WetA, SteC, MpkA, FadA, HymA*, and *SakA* genes. The *Brl A, Aba A*, and *Wet A* genes are pivotal to the central regulatory pathway of asexual sporulation in *A. nidulans*. The transcription levels of two of these pivotal sporulation-related genes, *AbaA* and *WetA*, were significantly downregulated in Δ*MrStuA* compared to those in the WT and C’ strains (*p* < 0.01) (**Figure 3**). In addition, the transcription levels of other sporulation related genes, including *MpkA, PhiA, MedA, AcoB, FluG*, and *FlbD*, were also downregulated. Meanwhile, the expression levels of other genes, such as *FlbA, BrlA, FlbC, SteC, Fad*A, *HymA*, and *SakA*, were not altered in the Δ*MrStuA* mutant. Collectively, the results indicate that the *MrStuA* gene regulates two key transcription factors, *AbaA* and *WetA*, that in turn impact a series of downstream sporulation-related genes at the molecular level. Therefore, *MrStuA* is necessary for normal sporulation in *M. robertsii*.

### Effect of *MrStuA* Function on Other Phenotypic Traits

**FIGURE 4:**
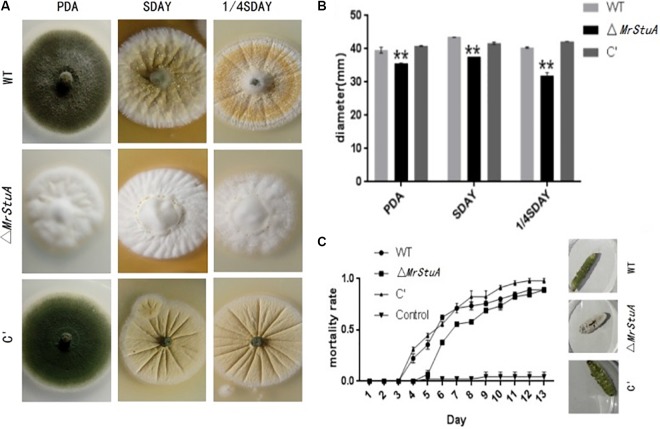
Effect of the *MrStuA* gene on other phenotypic traits. **(A)** Colonies from each strain grown on solid PDA, SDAY, and 1/4SDAY. **(B)** Radial growth rate of each strain on solid PDA, SDAY, and 1/4SDAY. **(C)** Virulence curves of each strain. Error bars are standard error of at least three independent experiments. Student’s *t*-test: ^∗^*p* < 0.05, ^∗∗^*p* < 0.01.

Morphological observations showed differences between Δ*MrStuA* colonies and those of the WT and C’ strains. On solid PDA, SDAY, and 1/4SDAY medium, the Δ*MrStuA* mutant produced white colonies, while the WT and C’ strains produced dark green colonies (**Figure 4A**). The radial growth of Δ*MrStuA* was significantly reduced compared to that of the WT and C’ strains (*p* < 0.05) under all growth conditions (**Figure 4B**).

In the UV treated group, the relative germination rates of the WT, Δ*MrStuA*, and C’ strains at 24 h were 62.55%, 63.38%, and 66.06%, respectively (**Supplementary Figure S4**). Under the 42°C water-bath treatment condition, the relative germination rates of the WT, Δ*MrStuA*, and C’ strains at 24 h were 76.77%, 81.95%, and 81.8%, respectively (**Supplementary Figure S5**). There were no significant differences in the tolerance of the germination of conidia to UV or heat treatments. Furthermore, there were no differences in the growth diameters of the WT, Δ*MrStuA*, and C’ strains on all chemical stress medium (**Supplementary Figures S2, S3**).

The Δ*MrStuA* strain exhibited reduced virulence compared to WT and C’ strains based on infection bioassays performed on the greater wax moth (*Galleria mellonella*). The Δ*MrStuA* strain (LT_50_ = 8.07 ± 0.68 d) took a longer time (19%) to kill the wax moths than the WT strain did (LT_50_ = 6.78 ± 0.77 d) (*P* < 0.05) (**Figure 4C**).

There were also differences in sporulation of the WT, Δ*MrStuA*, and C’ strains on host cadavers. The dead greater wax moths infected with the three *M. robertsii* strains were incubated at 25°C at 100% relative humidity for a week. Those killed by the WT and C’ strains produced numerous green spores; however, there were only white mycelia and a few conidia on the surface of host cadavers killed by the Δ*MrStuA* strain (**Figure 4C**).

Our results indicate that *MrStuA* may be involved in virulence and vegetative growth but has no impact on resistance to heat, UV, or chemical stress.

## Discussion

Proteins in the APSES family are regulatory transcription factors that exist ubiquitously in fungi. The functions of these proteins are related to sporulation, development, and the tolerance to unfavorable conditions (Doedt et al., 2004; Nishimura et al., 2009). *StuA* is a common member of Clade C-II of the APSES family; the function of *StuA* varies across different fungi. For example, *StuA* regulates sexual/asexual sporulation in *A. nidulans*, appressorium-mediated infection in *M. oryzae* (Doedt et al., 2004), two-phase conversion in *W. dermatitidis* (Wang and Szaniszlo, 2007), and secondary metabolites in *A. fumigatus* (Grosse et al., 2008). We found that in *M. robertsii, MrStuA* may play a crucial regulatory role in sporulation as well as minor roles in regulating virulence, germination, and vegetative growth.

The *M. robertsii* APSES transcription factor (EFZ01759) was identified as a C-II clade transcription factor based on a phylogenetic tree of APSES family gene data from different fungi. Some APSES family proteins are divided into the A-I, A-II, B, C-I, C-II, and D clades; at the same time, other APSES family genes, including XP_007819413, XP_007818991, XP_007821139, and EFY97406 from *M. robertsii*, belong to the A-I, A-II, B, and D clades, respectively. Thus, all five APSES protein family members are present in *M. robertsii*, demonstrating the richness and diversity of transcription factors of this family within *M. robertsii*. These APSES family proteins may play important roles in regulating the growth, differentiation, and infection processes in entomopathogenic fungi, especially for sporulation-related processes.

We found that the conidial yield from the Δ*MrstuA* mutant was 94.45% lower than the yield from the control strains. This observation is consistent with results from *A. nidulans* and *P. oxalicum*, as well as those from other fungi (Park and Yu, 2012). Microscopy revealed that the morphology of the conidia and sporulation structures of the mutant strains were significantly altered compared with those of the WT strains in some fungi (Mirabito et al., 1989; Prade and Timberlake, 1993). We found that in the *M. robertsii* mutant, the conidia became elongated, the sporulation became sparse, and the phialide became slender. However, the knockout of this gene in *A. nidulans* resulted in shortened conidiophores, reduced conidiophores, missing metulae and phialide, and a “stunted” phenotype. It has also been reported that there were no conidiophore structures when *StuA* was deleted in *P. oxalicum*. In addition, transcription levels of two central regulators of asexual development, *AbaA* and *WetA*, were significantly reduced in the Δ*MrstuA* mutant. These results indicate that *MrStuA* plays a regulatory role in some key genes, which in turn impact sporulation ability, conidia morphology, and sporulation structure. Thus, the gene *MrStuA* is required for sporulation.

The mutant strain has a significantly early germination time indicating that the *StuA* gene may be involved in regulating the germination process in *M. robertsii*. In *A. nidulans*, it has been reported that the *StuA* protein may regulate the temporal expression of genes (Adams et al., 1998). Thus, we speculate that this gene can impact the temporal expression of related genes.

The virulence of the mutant was significantly decreased, and the Δ*MrstuA* strain only produced a few conidia on the surface of host cadavers. The sporulation ability on host cadavers is also an important factor when determining insect virulence. In *M. acridum*, virulence decreased when a sporulation-related gene was knocked out; this effect is mainly due to the increased immunity of the host insect or the impact on its ability to form appressoria (Zhang et al., 2017). Thus, we conclude that the *MrStuA* gene is related to the virulence of *M. robertsii*.

In summary, the knock-out of the *MrStuA* gene led to significant changes in *M. robertsii* sporulation at both a phenotypic and molecular level; the deletion impacted virulence, germination, and vegetative growth. Thus, the *MrStuA* gene is involved in both infection and vegetative growth and is required for the sporulation process. It remains unclear how *MrStuA* regulates downstream genes, and further studies should focus on sporulation regulation pathways.

## Author Contributions

BH and ZW conceived and designed the study and edited the manuscript. WY wrote the manuscript, conducted the experiments, and analyzed the data. HW, QS, and LQ did a part of the experiments. BH supervised the project. All authors read and approved the final manuscript.

## Conflict of Interest Statement

The authors declare that the research was conducted in the absence of any commercial or financial relationships that could be construed as a potential conflict of interest.

## Footnotes

1 http://www.ncbi.nlm.nih.gov/

2 http://purl.org/phylo/treebase/phylows/study/TB2:S22030
