# Supplementary material for: The APSES Gene MrStuA Regulates Sporulation in Metarhizium robertsii
Source: Front Microbiol. 2018 Jun 5;9:1208. doi: 10.3389/fmicb.2018.01208 (PMC5996154; doi:10.3389/fmicb.2018.01208)
Supplement: Supplementary file 1 [file Data_Sheet_1.DOC]

Supplementary Material for ***Frontiers in Microbiology***

**Title:** The APSES family gene *MrStuA* regulates sporulation in ***Metarhizium robertsii***

**Authors:** WenjingYang a, HaoWu a, ZhangxunWang a,b,QianSun c, Lintao Qiao a, and Bo Huang a,*

**Affiliation:** a Anhui Provincial Key Laboratory of Microbial Pest Control, Anhui

Agricultural University, Hefei 230036, China

**Affiliation:**  b School of Plant Protection, Anhui Agricultural University, Hefei 230036,

China

**Affiliation:**  c Anhui SanLian University, Hefei 230036,China

*  **Corresponding author.** Bo Huang

Anhui Provincial Key Laboratory of Microbial Pest Control, Anhui Agricultural

University, Hefei 230036, China.

1. mail: bhuang@ahau.edu.cn Tel./ Fax: +86-551- 65786211.


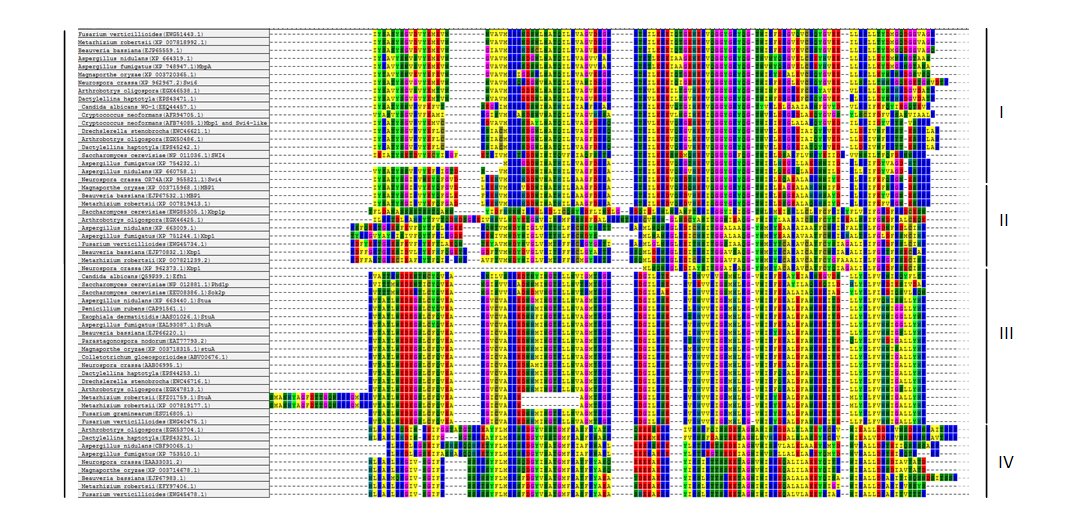


**Fig. S1. Multiple alignment of the APSES DNA-binding domain from different fungi**

**Table S1 Sequences of primers used for gene knockout,complement and identification**

| Gene | Primer 5’to 3’ | Sequence | Vector or purpose |
| --- | --- | --- | --- |
| Gene knockout |  |  |  |
| MrStuA | *MrStuA-F* | CTGGCTTGCCGTCTATTC | Verification |
|  | *MrStuA-R* | GCTTGAGGTCTCCGTTGC |  |
|  | *MrStuA*upF | G**GAATTC**GCTTTGTGCCTTGTATGTG | pDHt-bar |
|  | *MrStuA*upR | AA**CTGCAG**CGACAGGCGAGACAGAAT |  |
|  | *MrStuA*downF | GC**TCTAGA**ATCTTGCCACGCATCTCA |  |
|  | *MrStuA*downR | GC**TCTAGA**ACCGCAACGACTGGTGTAT |  |
| Bar | Bar-F | GGAGGTCAACAATGAATGCC | Verification |
|  | Bar-R | CCACGTCATGCCAGTTCC |  |
| Ben | Ben-F | GGTAACTCCACCGCCATCCA | Verification |
|  | Ben-R | GCAGGGTATTGCCTTTGGACTT |  |
| Gene complement |  |  |  |
| MrStuA | cp*MrStuA*F | G**ACTAGT**TGGAGCCCTAAGTCACAA | pDHt-ben |
|  | cp*MrStuA*R | GC**TCTAGA**CGTATTATTCGGGAGATGGT |  |

**Table S2 primers of conidiation related genes in *M.robertsii* for qRT-PCR**


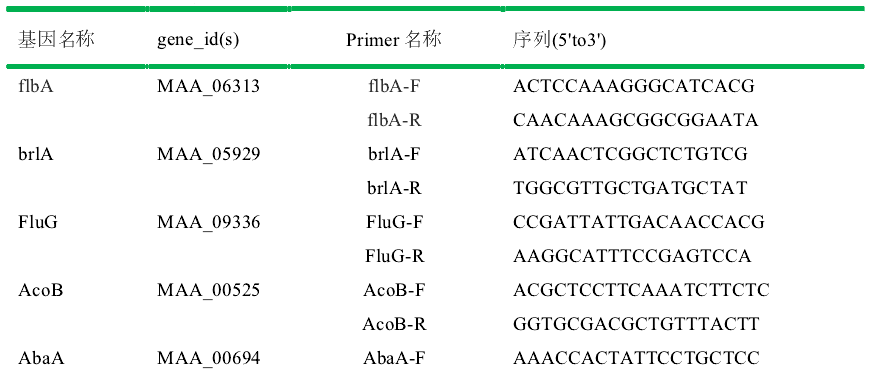


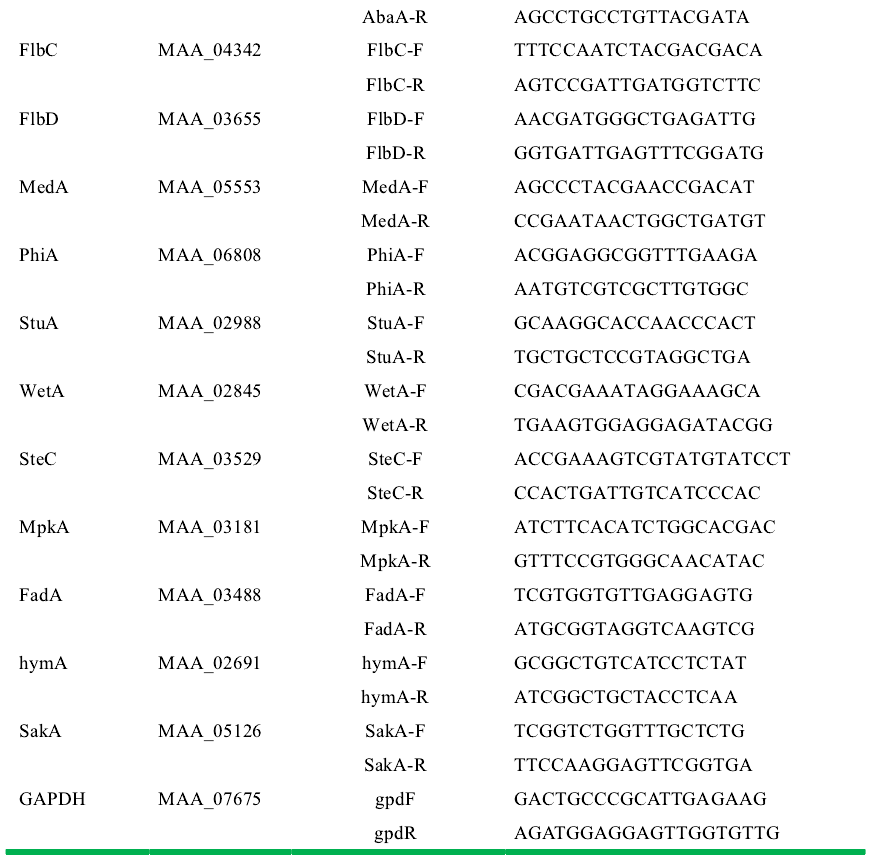


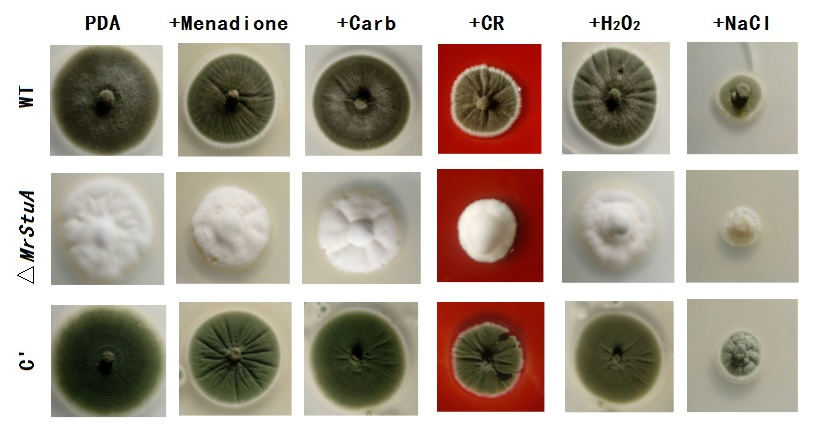


**Fig. S2. Stress responses mediated by *MrStuA***


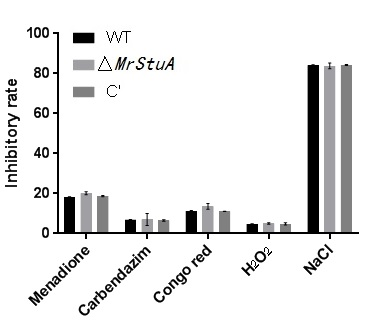


**Fig. S3. Inhibition of chemical drugs on diameter of △*MrStuA***

**
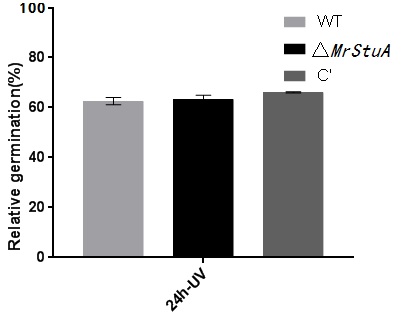
**

**Fig. S4. Mean relative percent germination of WT, △*MrStuA*, and C’conidia after exposure to UV radiation.**


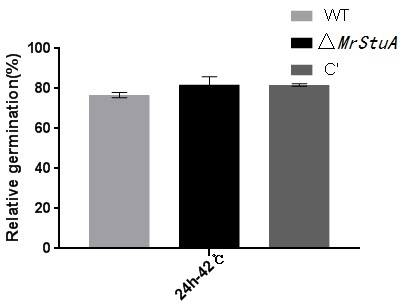


**Fig. S5.Mean relative percent germination of WT, △*MrStuA*, and C’conidia treated by heat**
